# Uncovering cell cycle-dependent effects on cell survival in near-infrared photoimmunotherapy

**DOI:** 10.1016/j.yexcr.2025.114570

**Published:** 2025-04-22

**Authors:** Atsushi Kaida, Yuriko Igarashi, Hitomi Nojima, Mio Nakayama, Ryuhei Okada, Ryosuke Takahashi, Hisataka Kobayashi, Masahiko Miura

**Affiliations:** a Department of Dental Radiology and Radiation Oncology, Graduate School of Medical and Dental Sciences, Institute of Science Tokyo, Japan; b Department of Oral and Maxillofacial Surgical Oncology, Graduate School of Medical and Dental Sciences, Institute of Science Tokyo, Japan; c Department of Head and Neck Surgery, Graduate School of Medical and Dental Sciences, Institute of Science Tokyo, Japan; d Molecular Imaging Branch, Center for Cancer Research, National Cancer Institute, National Institutes of Health, Bethesda, MD, 20892-1088, USA

**Keywords:** Near-infrared photoimmunotherapy, EGFR, Cell cycle, Cell survival, Head and neck cancer

## Abstract

Near-infrared photoimmunotherapy (NIR-PIT) is an innovative cancer treatment that selectively induces cell death in cancer cells. Cetuximab-IRdye700DX (Cmab-IR700) conjugate is commonly used in NIR-PIT for head and neck squamous cell carcinoma (HNSCC) because of the frequent overexpression of epidermal growth factor receptor (EGFR) in HNSCC cells. This study examined the influence of cell cycle phases on the response and sensitivity to NIR-PIT in cell lines expressing a fluorescent ubiquitination-based cell cycle indicator (Fucci). The timing of cell death was quantified using time-lapse imaging and a clonogenic assay was used to assess cell survival. The results indicated that the timing of cell death varied among cell lines, with G1-phase cells in HSC3 and CAL33 lines showing slower cell death than those in the S/G2/M phases, whereas HeLa cells exhibited no cell cycle phase-dependent correlation. Cell rupture was predominant in HSC3 and CAL33 cells, whereas HeLa cells exhibited a combination of cell rupture and swelling. Clonogenic survival differed among the cell lines, mirroring variations in the timing of cell death. Among CAL33 and HeLa cells, G1-phase cells demonstrated greater resistance to NIR-PIT. EGFR expression levels, which varied according to cell line and cell cycle phase, were associated with sensitivity to NIR-PIT. Additionally, L-ascorbic acid-treated HeLa cells exhibited increased time to cell death and reduced NIR-PIT sensitivity, which may be due to reactive oxygen species. These findings provide information for the development of NIR-PIT strategies based on cell cycle kinetics to enhance therapeutic outcomes.

## 1. Introduction

Near-infrared photoimmunotherapy (NIR-PIT) is an innovative cancer treatment that selectively induces cell death in cancer cells [1]. Therapy involves an antibody-photoabsorber conjugate (APC) that targets tumor-associated antigens on the cell surface [2]. Upon binding to tumor cells, near-infrared (NIR) light is applied to activate APC. In NIR-PIT, the photoabsorber used is IRdye700DX (IR700), which is a molecule with both hydrophobic and hydrophilic groups [3]. When exposed to NIR light, IR700 undergoes a structural change resulting in the loss of its hydrophilic group. This structural alteration damages the cell membrane, leading to an influx of extracellular fluid, subsequent cell swelling, and eventual cell rupture [4]. This form of cell death occurs rapidly after NIR-PIT. Importantly, the rupture of tumor cells releases antigens, which can activate the immune system, a phenomenon known as “immunogenic cell death” [5]. NIR-PIT has recently been applied clinically, particularly in patients with unresectable head and neck squamous cell carcinoma (HNSCC) [6,7]. The epidermal growth factor receptor (EGFR) is frequently overexpressed in HNSCC cells; therefore, a conjugate of cetuximab (a chimeric human-mouse anti--EGFR monoclonal antibody) and IR700 (Cmab-IR700) is used as an APC in this context [8]. Growing evidence supports the clinical effectiveness of EGFR-targeted NIR-PIT [6,7], suggesting that it may be a promising therapy for HNSCC. Despite an increasing understanding of the mechanisms underlying NIR-PIT [1,9] many aspects remain unclear.

Cell cycle kinetics are typically associated with the response to treatments, with radiosensitivity, for instance, varying according to the cell cycle phases [10–13]. Similarly, cell cycle phases can influence sensitivity to cancer treatments [14–17]. Therefore, cell cycle analysis provides valuable insights into the relationship between the cell cycle and therapeutic sensitivity. The fluorescent ubiquitination-based cell cycle indicator (Fucci) allows the determination of cell cycle phases based on fluorescent colors in live cells [18,19]. Fucci enables the analysis of various cell-cycle-related phenomena at the single-cell level. Specifically, cells with Fucci (SA) emitted red, orange (both red and green), and green fluorescence in the G1, early S, and S/G2/M phases, respectively. However, we can distinguish between G1, S, and G2/M phases in cells with Fucci (CA) because they emit red, green, and orange, respectively. Our previous studies using Fucci-expressing cells revealed associations between cell cycle phases and responses to treatments, such as ionizing radiation and drugs [13,20–23]. However, whether the cell cycle influences cell fate following NIR-PIT remains unknown. This study aimed to investigate the relationship between cell cycle phase, response, and sensitivity to NIR-PIT. Using Fucci-expressing cells, we demonstrated that NIR-PIT sensitivity varied by cell line and cell cycle phase owing to differential EGFR levels. Notably, HSC3 and CAL33 cells with high EGFR levels displayed immediate cell rupture after NIR-PIT, whereas HeLa cells with low EGFR levels exhibited delayed cell swelling. These findings may inform the development of optimized NIR-PIT therapeutic strategies.

## 2. Materials and methods

### 2.1. Cell lines

Human tongue squamous cell carcinoma cell lines, CAL33 and HSC3, and human cervical cancer cell line, HeLa, were used. Fucci (SA) was introduced into CAL33 and HSC3 cells. HeLa cells expressing Fucci (CA) 2 were provided by RIKEN BRC. Cells were cultured in DMEM containing high glucose (4500 mg/L) (Sigma-Aldrich, St. Louis, MO, USA) with 100 units/mL penicillin and 100 μg/mL streptomycin, supplemented with 10 % fetal bovine serum (FBS). The cells were incubated at 37 °C in a humidified 5 % CO_2_ atmosphere.

### 2.2. Reagents

Cetuximab (Cmab) was purchased from Merck Biopharma. IR700 NHS ester was obtained from Rakuten Medical. To prepare Cmab-IR700, Cmab was dialyzed and conjugated to IR700 as previously described [24]. L-ascorbic acid (L-Asc, A4403) was purchased from Sigma-Aldrich. L-Asc (100 mg) was dissolved in 5 mL of distilled water and used as a stock solution. To prepare the working solution, the stock solution was diluted with DMEM. The cells were incubated with 0.4 mM of L-Asc for 1 h before NIR-PIT. After NIR-PIT, L-Asc was maintained in the medium during time-lapse imaging or for 48 h in a clonogenic assay.

### 2.3. siRNA treatment

Lipofectamine^™^ RNAiMAX Transfection Reagent (Thermo Fisher Scientific, Waltham, MA, USA) was used to transfect 10 nM of siRNAs into cultured cells following the manufacturer’s protocol. For EGFR silencing, Silencer^™^ Select Validated siRNAs for human *EGFR* were purchased from Thermo Fisher Scientific, with siRNA sequences s564 (*EGFR*#1) and s565 (*EGFR*#2) used for knocking down *EGFR* expression. All experimental conditions included Stealth RNAi^™^ siRNA Negative Control Med GC Duplex #2 (Thermo Fisher Scientific, 12935112) as a non-targeting control to normalize for potential off-target effects.

### 2.4. Time-lapse imaging

A BIOREVO BZ-9000 or BZ-X800 fluorescence microscope (KEYENCE, Osaka, Japan) was used for time-lapse imaging. During imaging, cells were kept in an incubation chamber at 37 °C in a humidified atmosphere containing 95 % air and 5 % CO_2_ (Tokai Hit, Fujinomiya, Japan). Images were overlaid using a BZ-X Analyzer (KEYENCE). Pedigree analysis was performed using the time-lapse imaging data. Once the fluorescence color of a cell became unclear, cell death was defined. Time to cell death was defined as the time between the start of time-lapse imaging and cell death.

### 2.5. Clonogenic assay

Cells were trypsinized and treated with 10 μg/mL Cmab-IR700 for 60 min. Then, an appropriate number of cells in a single-cell suspension was exposed to NIR light (690 nm, 100 mW/cm^2^, 10 or 30 J/cm^2^) with an MLL-III-690 (Changchun New Industries Optoelectronics Tech, Changchun, China). Immediately after exposure to NIR light, the cells were plated in 6-well plates and incubated for 7–10 days. The colonies were fixed in 4 % paraformaldehyde and stained with 0.05 % crystal violet. Colonies comprising more than 50 cells were counted. The surviving fraction was calculated as the number of colonies divided by the number of cells seeded after exposure to NIR light at each dose, and then normalized to the plating efficiency.

### 2.6. Flow cytometric analysis

Briefly, cells were trypsinized and centrifuged. After washing the resulting cell pellets in PBS, cells were treated with 10 μg/mL Cmab-IR700 for 60 min. Finally, the treated cells were filtered through a nylon mesh to obtain a single-cell suspension. Samples were analyzed using a FACSAria III or FACSLyric flow cytometer (BD Bioscience, Franklin Lakes, NJ, USA) and FlowJo software (BD Bioscience). The median was calculated for each dataset, and the overall mean and standard error were derived from the median values across at least three independent experiments.

### 2.7. Cell sorting

A FACSAria III cell sorter (BD Bioscience) was used to isolate cells in each cell cycle phase according to Fucci fluorescence. To prevent cells from shifting in the cell cycle during sorting, the cells were maintained below 4 °C, and all experiments, from cell sorting to NIR light exposure, were performed within an hour.

### 2.8. Statistical analysis

Differences in the time to cell death, surviving fractions between cell cycle phases, and relative EGFR expression were analyzed using either one-way ANOVA with Tukey’s multiple comparison test, or the Kruskal-Wallis test with Dunn’s multiple comparison test. Differences in the surviving fractions between cell lines were analyzed using two-way ANOVA with Sidak’s multiple comparison test. Statistical analyses were performed using the GraphPad Prism software (GraphPad Software, San Diego, CA, USA). *P* values < 0.05 were considered statistically significant.

## 3. Results

### 3.1. Differences in timing of cell death after NIR-PIT according to cell lines and cell cycle phases

**Fig. 1.**
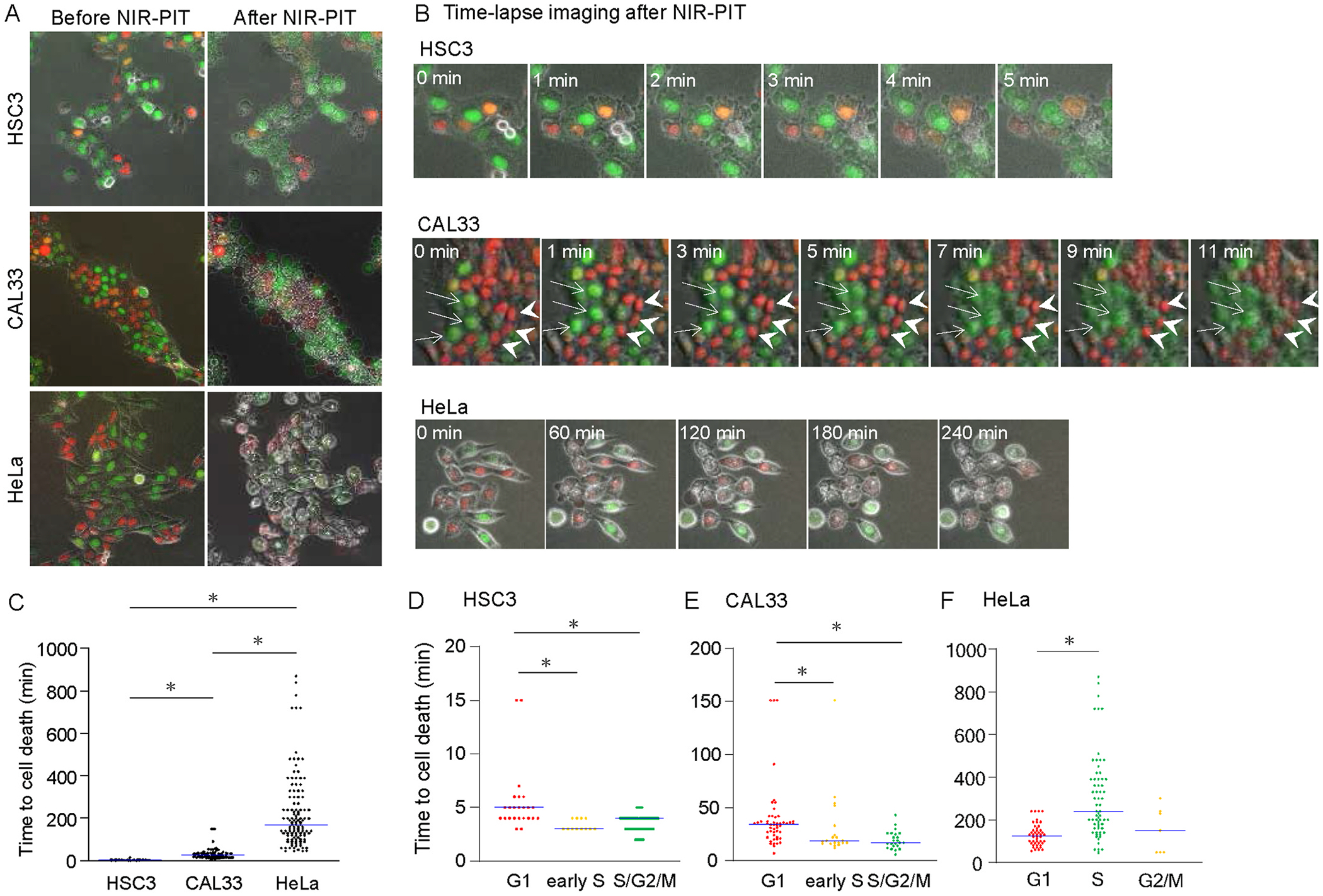
Differences in timing of cell death after NIR-PIT according to cell lines and cell cycle phases **A**. Representative images of HSC3, CAL33, and HeLa cells expressing Fucci before and after NIR-PIT. **B.** Time-lapse imaging of HSC3, CAL33, and HeLa cells expressing Fucci after NIR-PIT. Indicated time shows time after NIR-PIT. Zero min indicates the time before NIR-PIT. In CAL33 cells, arrows and arrowhead represent green and red cells, respectively, before NIR-PIT. **C.** Quantitative analysis of the timing of cell death after NIR-PIT in HSC3, CAL33, and HeLa cells. When the Fucci fluorescence became unclear, we defined it as cell death. The data shown are represented as plots, for which black dots correspond to individual cells and the horizontal solid line shows the median. **D-F.** Quantitative analysis of the timing of cell death after NIR-PIT according to cell cycle phases in HSC3 (D), CAL33 (E), and HeLa (F) cells. HSC3 and CAL33 cells expressed Fucci (SA), while HeLa cells expressed Fucci (CA)2. In these assays, cells were treated with 10 μg/mL Cmab-IR700 for 60 min. After washing cells, cells were irradiated with NIR light at 10 J/cm^2^. Cells at each cell cycle phase were sorted according to the Fucci fluorescence at the start of time-lapse imaging. The data shown are represented as plots, for which each color of dots correspond to individual cells at each cell cycle phase and the horizontal solid line shows the median. *P* value was calculated by the Kruskal-Wallis test.

NIR-PIT induces cell death immediately after exposure to NIR light when an APC binds to an antigen on the cell surface. Time-lapse imaging of HSC3, CAL33, and HeLa cells was performed to determine whether this was a universal phenomenon in cell lines. Cell death was observed after NIR-PIT treatment in all three cell lines. Specifically, all of the cells exhibited cell rupture, a representative pattern of cell death after NIR-PIT, in HSC3 and CAL33 cells (Fig. 1A and B; HSC3 and CAL33). In HeLa cells, a different type of cell death was observed in addition to cell rupture (Fig. 1A and B, HeLa). We noticed that the timing of cell death differed between cell lines. The timing of cell death was measured based on time-lapse imaging results. In the HSC3 cells, cell death was rapidly induced after NIR-PIT, with most cells dying within 5 min (Fig. 1C). CAL33 cells also exhibited early cell death after NIR-PIT, although the induction of cell death took longer than in HSC3 cells. However, HeLa cells were resistant to NIR-PIT, and cell death did not occur immediately after NIR-PIT. Therefore, the timing and pattern of cell death after NIR-PIT varied among the three cell lines tested.

Cells expressing Fucci allowed us to distinguish between the different cell cycle phases based on the fluorescence color. The cell populations were grouped according to the cell cycle phases at the start of imaging and the timing of cell death was analyzed for each phase. In detailed observation, we noticed that cell death could be observed more rapidly in green cells, compared to red cells (Fig. 1B, CAL33). Indeed, quantification revealed that cell death occurred quickly in cells exposed to NIR light in the S/G2/M phases, compared to HSC3 and CAL33 cells in the G1 phase (Fig. 1D and E), while we did not observe the same tendency in HeLa cells (Fig. 1F). Thus, these results suggest that HSC3 and CAL33 cells showed rapid cell death, especially in the S/G2/M phase, although HeLa cells differed from the other two cell lines in the timing of cell death and cell cycle phase dependency.

### 3.2. Effects of NIR-PIT on cellular clonogenicity

**Fig. 2.**
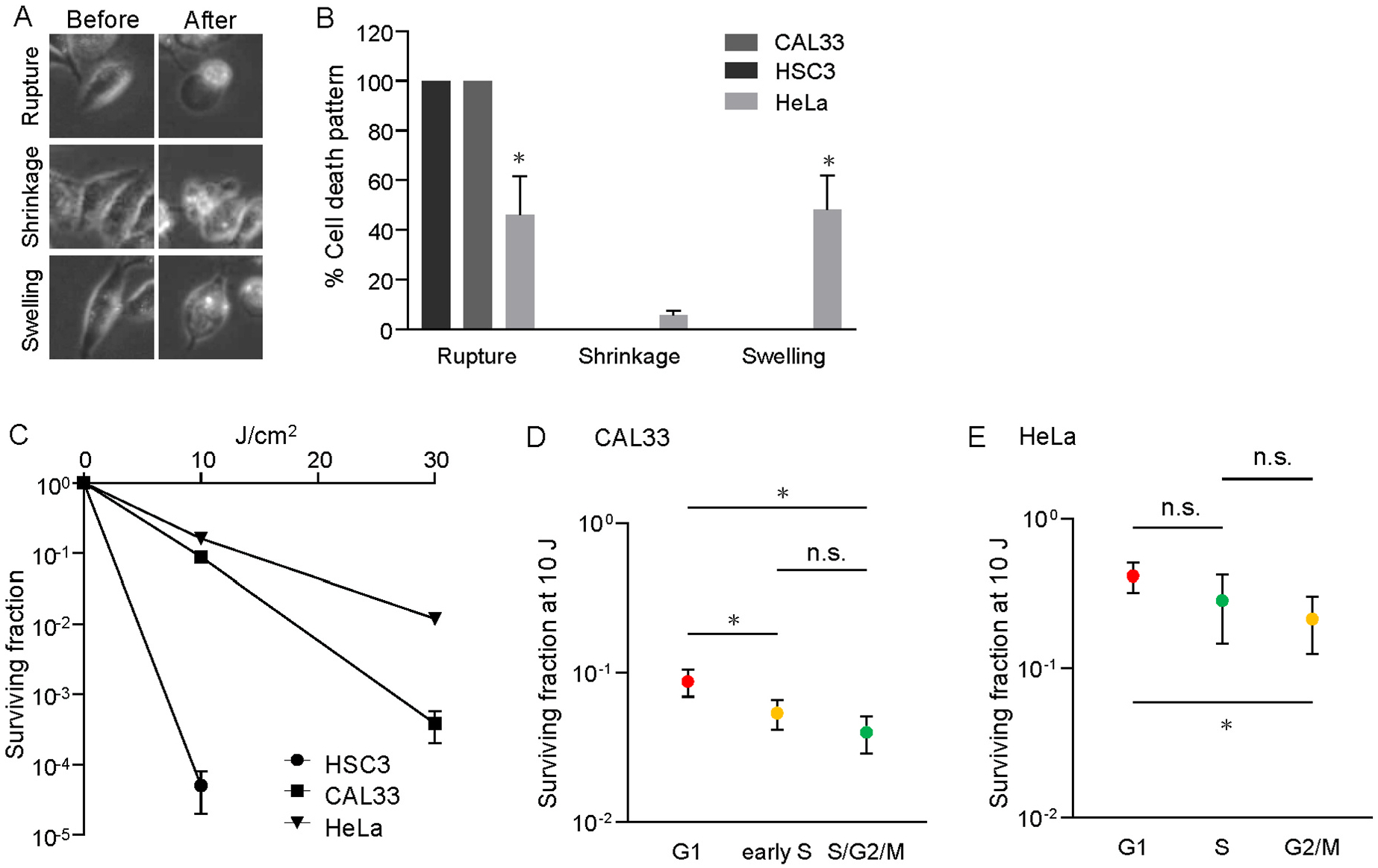
Effects of NIR-PIT on cellular clonogenicity **A.** Representative images of major types of cell death, cell rupture (rupture), shrinkage, and cell swelling without cell rupture (swelling), after NIR-PIT in HeLa cells. **B.** Quantitative analysis of the types of cell death after NIR-PIT in HSC3, CAL33, and HeLa cells. **C.** Survival curves after NIR-PIT in HSC3, CAL33, and HeLa cells. Plots and error bars indicate mean and standard error (SE), respectively. Some error bars are hindered by plots due to small SE. **D&E**. Comparison of surviving fractions after NIR-PIT between cell cycle phases in CAL33 (D) and HeLa (E) cells. Plots and error bars indicate mean and standard error (SE), respectively. In these assays, cells were irradiated with NIR light at 0, 10, or 30 J/cm^2^ after treatment with 10 μg/mL Cmab-IR700 for 60 min. At least 50 cells were counted. At least three independent experiments were performed. *P* value was calculated by the two-way (B) or one-way (D&E) ANOVA.

In general, NIR-PIT induces cell rupture, a significant characteristic of cell death, leading to the release of intracellular contents [1]. We clearly observed this pattern in HSC3 and CAL33 cells, whereas HeLa cells underwent different mechanisms of cell death after NIR-PIT, including cell shrinkage and cell swelling without cell rupture, in addition to cell rupture (Fig. 2A). Quantification revealed decreased cell rupture but also increased cell swelling in HeLa cells. In contrast, both HSC3 and CAL33 cells exhibited cell rupture in all instances. (Fig. 2B). Although previous studies have revealed rapid induction of cell death after NIR-PIT [25,26] there have been no reports demonstrating clonogenicity after NIR-PIT. A colony formation assay was performed to investigate the effect of NIR-PIT on clonogenicity. Although we expected that no colonies would be formed after NIR-PIT because of its lethality, the clonogenic assay revealed colony-forming potential after NIR-PIT (Fig. 2C, Supplementary Fig. 1). The surviving fraction decreased in a dose-dependent manner in all the cell lines. Among the cell lines used in this study, HSC3 cells showed the highest sensitivity to NIR-PIT, whereas HeLa cells were less sensitive than HSC3 and CAL33 cells (Fig. 2C). This difference in clonogenicity between cell lines correlated with the timing of cell death.

Next, we examined the effect of cell cycle phases on clonogenicity after NIR-PIT by isolating cells at each cell cycle phase with a cell sorter based on Fucci fluorescence. As HSC3 cells were too sensitive to NIR-PIT even at low doses, we used CAL33 and HeLa cells for this assay. In both the CAL33 and HeLa cell lines, the surviving fraction was significantly higher in G1 cells than in S/G2/M and G2/M cells (Fig. 2D and E). This result also correlated with the timing of cell death in CAL33 cells but not in HeLa cells. Thus, these findings suggested that the colony-forming potential differed according to the cell line, which was correlated with the timing of cell death after NIR-PIT. In addition, S/G2/M cells were more sensitive to NIR-PIT than cells in earlier phases in both cell lines, although the sensitivity was not always dependent on the timing of cell death, as observed in HeLa cells.

### 3.3. Differential expression of EGFR between cell lines and cell cycle phases

**Fig. 3.**
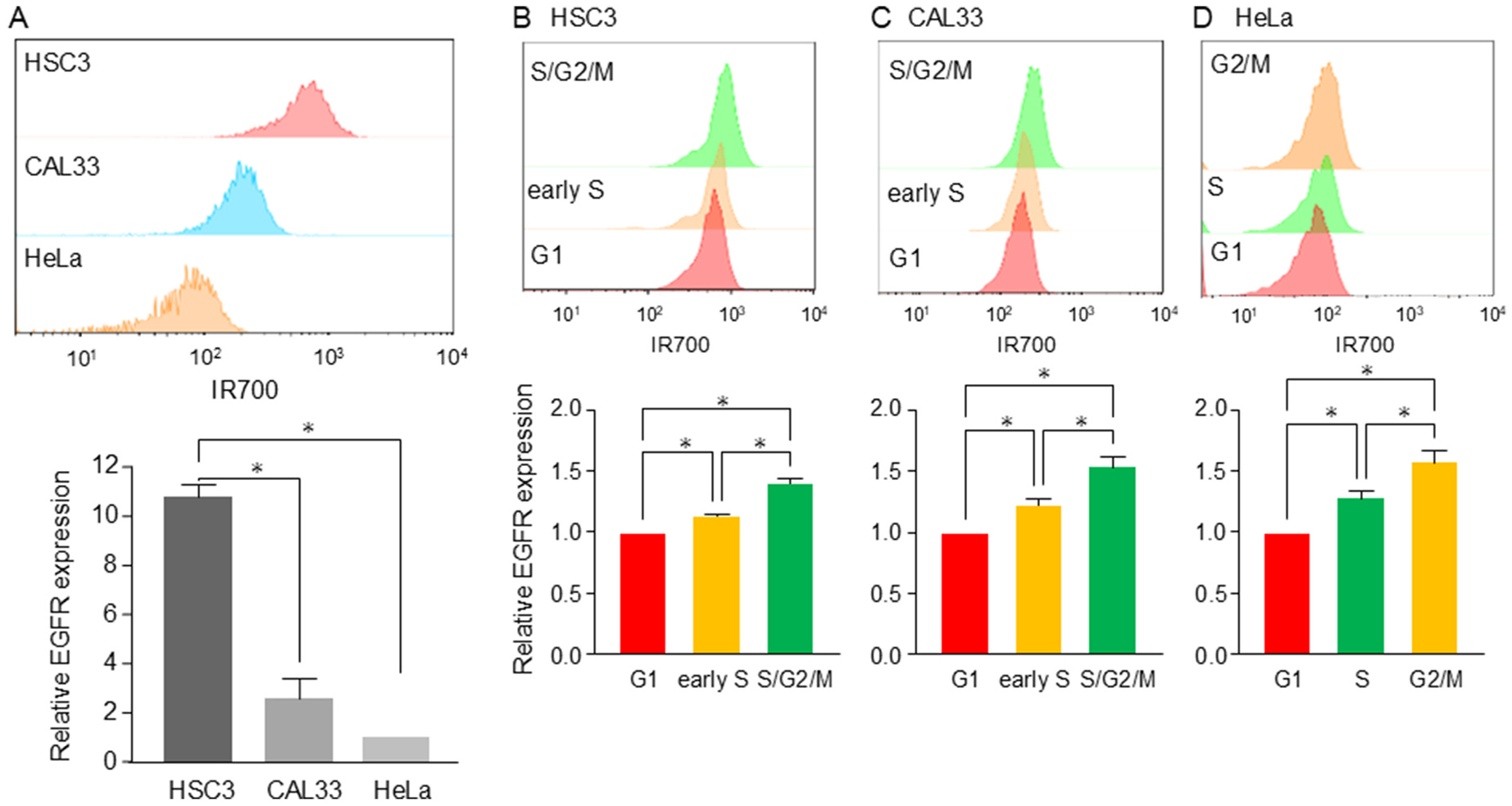
Differential expression of EGFR between cell lines and cell cycle phases **A.** Comparison of EGFR levels between HSC3, CAL33, and HeLa cells. Representative histograms are shown (top). Each expression level was normalized with the signal intensity of HeLa cells. A bar graph and error bars show mean and SE of relative EGFR expression levels (bottom), respectively. **B-D.** Comparison of EGFR expression levels between cell cycle phases in HSC3 (B), CAL33 (C), and HeLa (D) cells. Representative histograms are shown (top). Each expression level was normalized with the signal intensity of G1 cells. A bar graph and error bars show the mean and SE of relative EGFR expression levels (bottom), respectively. At least three independent experiments were performed. *P* value was calculated by the one-way ANOVA.

In general, after Cmab-IR700 binds to EGFR, exposure to NIR light induces damage on the cell surface, resulting in cell death. The extent of cell damage may depend on the binding potential of Cmab-IR700 to EGFR. Subsequently, we hypothesized that the differences in response and sensitivity to NIR-PIT might be due to the difference in its binding potential. We analyzed the binding levels of Cmab-IR700 to EGFR in each cell line by FACS. As shown in Fig. 3A, the binding levels in HSC3 cells were higher than those in CAL33 and HeLa cells. HeLa cells exhibited the lowest binding levels among all the cell lines. This result was consistent with the response and sensitivity to NIR-PIT. In addition, we separated the cell populations according to Fucci fluorescence and examined their binding potential to EGFR in each cell cycle phase (Fig. 3B–D). Intriguingly, the binding levels were lowest in the G1 phase, whereas cells in the S/G2/M phase showed higher levels of EGFR in the three cell lines. Although this result was not consistent with the response in HeLa cells alone, the sensitivity correlated with the difference in EGFR levels between the cell cycle phases.

Given that EGFR protein levels are critical for determining the cellular response to NIR-PIT, EGFR knockdown could significantly alter treatment effectiveness. We performed siRNA-mediated silencing to downregulate EGFR expression and examined whether EGFR levels correlated with the response to NIR-PIT in HSC3 and CAL33 cells. Two distinct siRNAs targeting *EGFR* (*EGFR*# 1 and *EGFR*# 2) effectively decreased EGFR protein levels (Supplementary Fig. 2A). Consistently, the binding levels of Cmab-IR700 to EGFR were significantly reduced by these *EGFR*-targeting siRNAs in both HSC3 and CAL33 cells compared with the negative control siRNA (Supplementary Fig. 2B and C). As expected, cell death was not observed within a short time period (~20 min) after NIR-PIT when HSC3 and CAL33 cells were pretreated with *EGFR*-targeting siRNAs (Supplementary Fig. 2D). Further long-term observation for 24 h confirmed the substantially reduced cytotoxicity of NIR-PIT in *EGFR*-knockdown HSC3 and CAL33 cells. These findings clearly demonstrate that cellular response to NIR-PIT is largely dependent on the EGFR expression levels.

### 3.4. Inhibitory effect of L-ascorbic acid on NIR-PIT-induced cell death in HeLa cells

**Fig. 4.**
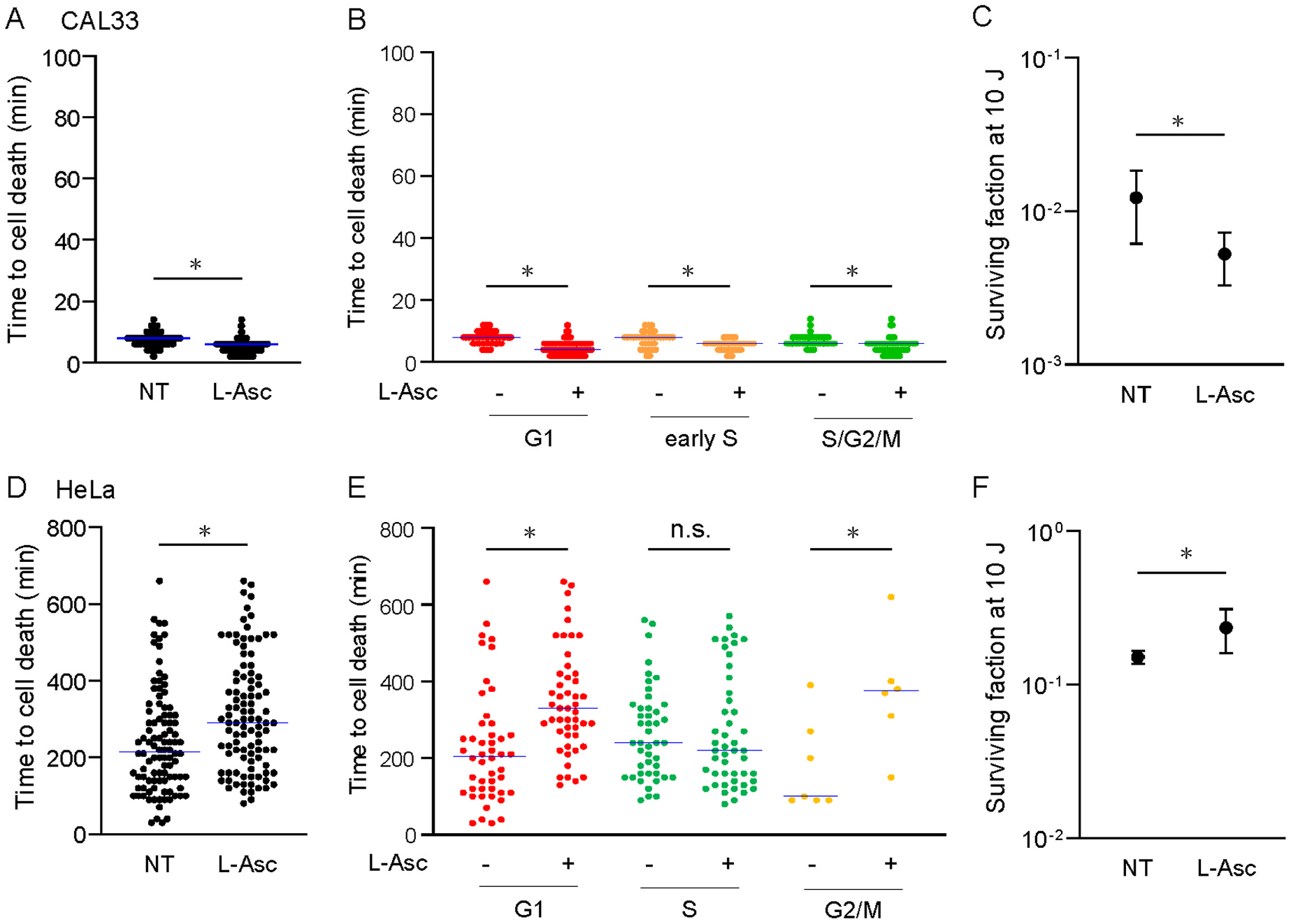
Inhibitory effect of L-ascorbic acid on NIR-PIT-induced cell death in HeLa cells **A&D.** Quantitative analysis of the timing of cell death after NIR-PIT with or without 0.4 mM L-ascorbic acid (L-Asc) in CAL33 (A) and HeLa (D) cells. **B&E.** Quantitative analysis of the timing of cell death after NIR-PIT with or without 0.4 mM L- Asc in CAL33 (B) and HeLa (E) cells. All traced cells were analyzed in A or D, and based on the results, cells at each cell cycle phase were sorted according to Fucci fluorescence at the start of time-lapse imaging. The data shown are represented as plots, in which dots correspond to individual cells, and the horizontal solid line shows the median (A, B, D, and E). **C&F.** Comparison of cell survival after NIR-PIT with or without 0.4 mM L- Asc in CAL33 (C) and HeLa (F) cells. The plots and error bars represent the mean and SE. In these assays, cells were irradiated with NIR light at 10 J/cm^2^ after treatment with 10 μg/mL Cmab-IR700 for 60 min. L-Asc was applied together with Cmab-IR700, and the cells were exposed to L-Asc during time-lapse imaging or for 48 h in a clonogenic assay. *P* value were calculated using the Mann-Whitney *U* test (A&D), Kruskal-Wallis test (B&E), or two-tailed Student’s t-test (C&F).

We observed a differential response to NIR-PIT in these cell lines. In particular, HeLa cells were resistant to NIR-PIT in terms of the timing of cell death and clonogenicity compared to the other cell lines (Figs. 1 and 2). Furthermore, cell swelling, bleb formation, and rupture were observed immediately after NIR-PIT in HSC3 and CAL33 cells, whereas different cell death pattern was detected later in HeLa cells. (Fig. 2A). Indeed, a previous study revealed that NIR-PIT rapidly induces damage to the cell membrane and generates singlet oxygen and reactive oxygen species (ROS) [9]. These findings suggested that different mechanisms underlie cell death in HeLa cells. We examined the effect of the anti-oxidant L-ascorbic acid (L-Asc) in response to NIR-PIT. In CAL33 cells, which showed rapid cell death, L-Asc further accelerated cell death, irrespective of the cell cycle phase (Fig. 4A and B). Sensitivity to NIR-PIT also increased with L-Asc treatment (Fig. 4C). In contrast, the addition of L-Asc increased the time required for HeLa cell death (Fig. 4D). When the results were further analyzed according to the cell cycle phase, the G1 and G2/M phases, but not the S phase, were affected by L-Asc (Fig. 4E). Moreover, L-Asc contributed to increased resistance to NIR-PIT in HeLa cells (Fig. 4F). These findings suggest that membrane damage as well as singlet oxygen and ROS levels may at least partially contribute to cell death in HeLa cells.

### 3.5. Changes in cell cycle kinetics in survival HeLa cells after NIR-PIT

**Fig. 5.**
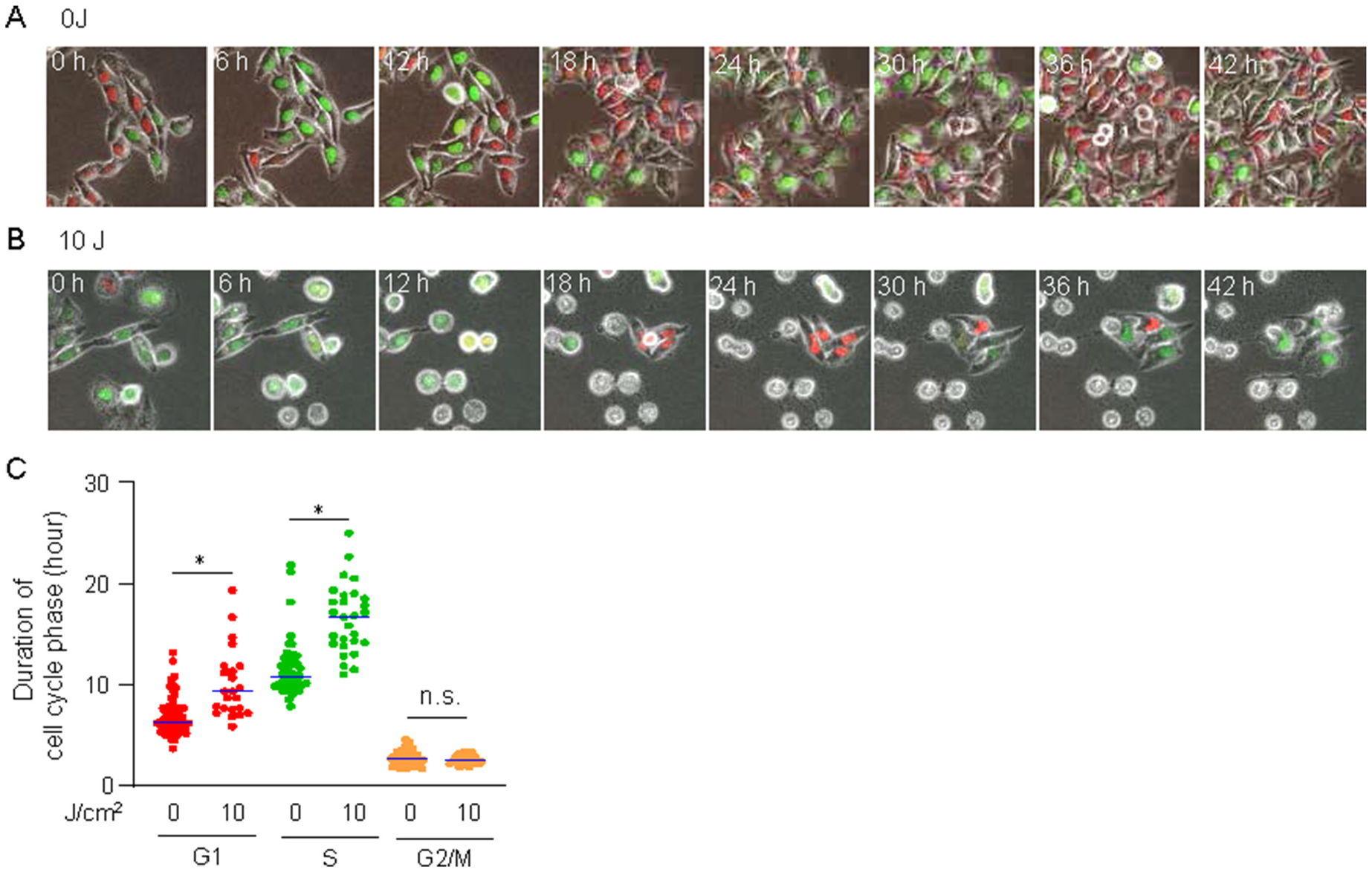
Changes in cell cycle kinetics in survival HeLa cells after NIR-PIT **A&B.** Time-lapse imaging of HeLa cells after NIR-PIT at 0 J and 10 J At 0 J, cells were treated with Cmab-IR700 alone. At 10 J, representative images of the dead and surviving cells were obtained. The indicated time indicates the time after NIR-PIT. Zero h indicates the time before NIR-PIT. **C.** Quantitative analysis of the duration of each cell cycle phase in the surviving cells after NIR-PIT. The data are represented as plots, for which the dots correspond to individual cells and the horizontal solid line shows the median. A minimum of 50 cells were counted. At least three independent experiments were conducted. *P* value were calculated using the Kruskal-Wallis test.

Our clonogenic assay highlighted the potential for cell survival after NIR-PIT, particularly in HeLa cells (Fig. 2C), suggesting that HeLa cells continued to grow after NIR-PIT, which could lead to tumor recurrence. To explore the cell cycle dynamics in these surviving cells, we conducted time-lapse imaging and identified a few surviving cells that appeared to proliferate throughout the imaging period. Notably, the cell cycle duration was prolonged (Fig. 5A and B). We quantified the duration of the G1, S, and G2/M phases (Fig. 5C) and found that the irradiated cells had significantly extended cell cycles compared to those treated with Cmab-IR700 alone, with pronounced effects in the G1 and S phases. These findings indicate that, although cells can survive and regrow post-NIR-PIT, cell cycle progression is notably altered.

## 4. Discussion

This study investigated the cell kinetics and cell cycle-dependent effects of NIR-PIT using Cmab-IR700, specifically focusing on the relationship between cell cycle phases, EGFR expression, and cell death in multiple cell lines. Effective induction of cell death in NIR-PIT requires EGFR expression on the cell surface, as Cmab binds to EGFR to deliver the IR700 conjugate. Flow cytometric analysis revealed distinct EGFR expression levels across cell lines, which correlated with the cellular response and sensitivity after NIR-PIT. In HSC3 cells, which had the highest EGFR expression among the cell lines tested, we observed rapid cell death after NIR-PIT, reflecting high sensitivity to the treatment. Conversely, HeLa cells, which express low levels of EGFR, exhibit a delayed cell death response that correlates with reduced NIR-PIT sensitivity. CAL33 cells, with intermediate EGFR expression, showed responses similar to those of HSC3 cells, but were distinct from those of HeLa cells. In addition, EGFR knockdown by siRNA significantly rendered cells resistant to NIR-PIT, confirming the critical role of EGFR in mediating NIR-PIT efficacy. These findings are consistent with those of previous studies investigating the effect of EGFR expression on NIR-PIT cytotoxicity, particularly its dependence on EGFR levels [26,27]. Furthermore, these results allowed us to classify cell lines into two groups based on the timing of cell death following NIR-PIT: an “immediate cell death” group (HSC3 and CAL33) and a “delayed cell death” group (HeLa). Consistent with prior studies [1,26,27], NIR-PIT induced rapid cell death through membrane damage characterized by swelling, rupture, and immediate cell death in HSC3 and CAL33 cells. However, we observed a distinct pattern of cell death in HeLa cells, potentially due to ROS production. NIR-PIT generates singlet oxygen and ROS [9]. NIR-PIT-induced cytotoxicity was inhibited under hypoxia and by reducing agents, including L-Asc and L-cysteine, although the inhibitory effect depended on drug concentration. Supporting this, the addition of L-Asc delayed cell death and increased cell survival in HeLa cells, while paradoxically enhancing NIR-PIT sensitivity in CAL33 cells, which may be due to the anti-cancer effects of L-Asc [28–30].

Using Fucci-expressing cell lines, we demonstrated for the first time that EGFR levels fluctuate with cell cycle phase, with notably higher EGFR levels observed in the S/G2 phase than in the G1 phase. EGFR expression levels vary with change in the cell cycle phase, resulting in cell cycle-dependent timing of cell death and survival in HSC3 and CAL33 cells. Although HeLa cells also display cell cycle phase-dependent EGFR differences, no correlation was found between cell cycle phases and cell death in these cells. This observation suggests that changes in EGFR expression across the cell cycle phases are a universal phenomenon. While there are no direct studies linking EGFR levels to cell cycle phases, previous studies have demonstrated cell cycle-dependent changes in plasma membrane structure [31–33]. Furthermore, the mammalian cell size increases as cells progress through the cell cycle [34–36], with cells typically being larger in the S and G2 phases than in G1 phase. As the cell size increases, the surface area and number of cell surface receptors may increase. Although no studies have directly linked cell size to receptor levels, nanoparticle uptake has been shown to be correlated with cell size [37]. These findings suggest that cell size-and cell cycle phase-dependent factors may influence EGFR expression. Further studies are necessary to clarify the mechanisms underlying the cell cycle-dependent regulation of EGFR.

We performed the first clonogenic assay with NIR-PIT, revealing the presence of surviving cells post-treatment, particularly HeLa cells with low EGFR expression levels. Time-lapse imaging revealed clusters of surviving cells exhibiting atypical cell death patterns, suggesting an alternative mechanism involving ROS generation. Furthermore, siRNA-induced EGFR depletion clearly nullified the therapeutic efficacy of NIR-PIT in combination with Cmab-IR700, suggesting that EGFR expression levels could serve as a predictive biomarker for treatment response. Solid tumors are often characterized by cellular heterogeneity and diverse tumor microenvironments [38,39] which may influence the EGFR expression levels in individual tumor cells. Given that tumor cells with varying EGFR levels exist within solid tumors, a subpopulation with low EGFR expression may survive NIR-PIT treatment. This suggests that these cells retain their clonogenic potential, potentially acting as the origin of recurrence after NIR-PIT. Interestingly, during imaging, surviving HeLa cells displayed prolonged G1 and S phases compared with non-irradiated cells, indicating that NIR-PIT may affect cell cycle progression. While typical cell cycle arrest results from DNA damage-activated checkpoints [40], NIR-PIT primarily targets the cell membrane rather than DNA [3–5]. We consider two potential reasons for the observed cycle delay. First, NIR-PIT-induced ROS production may be a contributing factor. Our findings in HeLa cells indicated the presence of ROS after treatment, potentially delaying cell death compared to other cell lines. ROS can damage DNA, potentially activating checkpoints and arresting the cycle [41]. Second, cell membrane damage may activate cell cycle checkpoints—a non-canonical pathway–but one with some support from studies linking membrane stress to cell cycle arrest [42–44]. Additionally, our data indicate that prolongation of the G1 phase significantly contributes to cell cycle delay. In general, HeLa cells with non-functional p53 (due to HPV infection) bypass G1 arrest following DNA damage because the G1/S checkpoint relies on functional p53 [20,21]. However, evidence shows that p21 and p27 can induce G1 arrest, regardless of p53 status, in response to certain drugs [45–47]. The prolonged G1 phase after NIR-PIT may result from another mechanism independent of p53 function. Thus, prolonged G1 phase in HeLa cells post-NIR-PIT may occur via p53-independent mechanisms. This cell cycle delay likely provides time for NIR-PIT-induced damage repair, potentially enabling these cells to resume proliferation. Because these surviving cells may drive recurrence or metastasis, future research is essential to understand the underlying mechanisms and develop strategies that specifically target these surviving cell populations.

This study had several limitations. First, we used three different cell lines and observed differential EGFR expression levels, with HeLa cells (derived from human cervical cancer) showing lower EGFR levels than HSC3 and CAL33 cells (derived from human oral cancer). Differences in cell death kinetics following NIR-PIT treatment were strongly correlated with EGFR expression levels across the cell lines tested. EGFR knockdown significantly attenuated NIR-PIT-induced cell death in both HSC3 and CAL33 cells, providing compelling evidence that the cytotoxic effects of NIR-PIT are directly dependent on EGFR expression. Additionally, the cellular origin may have influenced the observed patterns of cell death. Notably, siRNA-mediated EGFR knockdown reduced receptor levels below those naturally occurring in HeLa cells (data not shown), and consequently, no cell death was observed upon NIR-PIT treatment under these conditions. This finding suggests a potential threshold of EGFR expression required for effective NIR-PIT induced cytotoxicity. Further investigations are necessary to determine whether atypical forms of cell death observed in HeLa cells also occur in HNSCC cells with inherently low EGFR expression or following induced EGFR down-regulation. Second, although we observed cell cycle-dependent cytotoxicity in HSC3 and CAL33 cells *in vitro*, this effect was not tested *in vivo*. Dormant cells in solid tumors, often in the G1/G0 phase due to tumor microenvironmental factors, such as hypoxia, acidity, and nutrient scarcity [38,39], may display higher resistance to NIR-PIT, given their lower EGFR expression in G1. Thus, further *in vivo* studies are essential to identify the origin of recurrence following NIR-PIT. In conclusion, our findings provide the first evidence that cell survival after NIR-PIT is influenced by EGFR levels and cell cycle phase, and underscore the need to consider these factors when optimizing NIR-PIT therapeutic strategies.

## Supplementary Material

1

## Data Availability

Data will be made available on request.

## Appendix A. Supplementary data

Supplementary data to this article can be found online at https://doi.org/10.1016/j.yexcr.2025.114570.

## Abbreviations:

APC: antibody-photoabsorber conjugate
EGFR: epidermal growth factor receptor
FBS: fetal bovine serum
Fucci: fluorescent ubiquitination-based cell cycle indicator
HNSCC: head and neck squamous cell carcinoma
NIR: near-infrared
NIR-PIT: near-infrared photoimmunotherapy
