# Supplementary material for: Uncovering cell cycle-dependent effects on cell survival in near-infrared photoimmunotherapy
Source: Exp Cell Res. Author manuscript; Available in PMC 2026 May 19. (PMC13184561; doi:10.1016/j.yexcr.2025.114570)
Supplement: 1 [file NIHMS2174809-supplement-1.pdf]

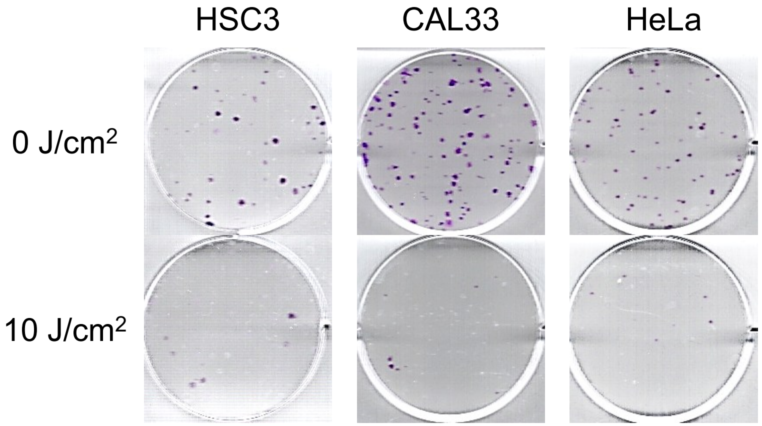

Supplementary Fig.1 Representative images of the clonogenic assay following NIR-PIT treatment.
Precise cell numbers were optimized for each cell line and irridation dose as follows: HSC3 (0 J/cm^2-300 cells, 10 J/cm^2-5$\times$10^4 cells); CAL33 (0 J/cm^2-400 cells, 10 J/cm^2-4000 cells); HeLa (0 J/cm^2-100 cells, 10 J/cm^2-1000 cells). Cells were plated immediately after exposure to NIR light and cultured until colony formation.

Supplementary Fig.2

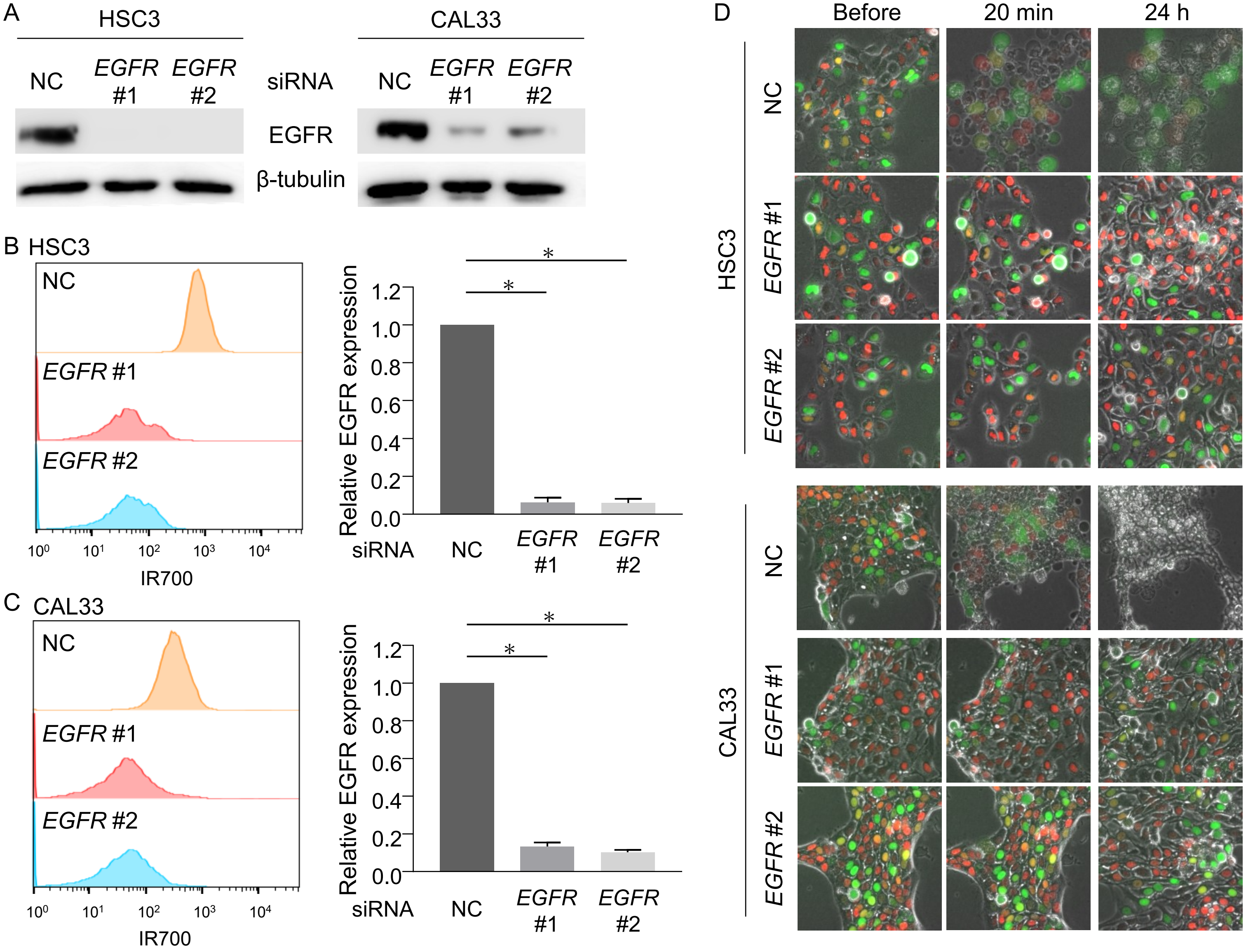

Supplementary Fig.2 Attenuated NIR-PIT cytotoxicity in *EGFR*-knockdown HNSCC cell lines

A. Western blot analysis for EGFR and β-tubulin (loading control) expression in HSC3 (left) and CAL33 (right) cells transfected with negative control (NC) and *EGFR*-targeting siRNAs (EGFR#1 and EGFR#2). **B** and **C**. Representative flow cytometry histograms (left) and quantitative analyses (right) of *EGFR* expression in negative control and EGFR-knockdown HSC3 (B) and CAL33 (C) cells. Expression level were normalized to the signal intensity of negative control cells. Bar graph display mean relative EGFR expression levels with standard error (SE) indicated by error bars . **D**. Representative fluorescence microsopy images of Fucci-expressing HSC3 (top) and CAL33 (bottom) cells transfected with negative control or *EGFR*-targeting siRNAs (*EGFR* #1 and #2) before and after NIR-PIT treatment. Time point relative to NIR-PIT application are indicated above each image. All experiments were performed in at least three independent replicates. Statistical significance was determined by one-way ANOVA (*$P < 0.05$)
